# Assessment of water enema PET/CT: an effective imaging technique for the diagnosis of incidental colorectal ^18^F-FDG uptake

**DOI:** 10.1186/s12880-023-01186-0

**Published:** 2024-01-03

**Authors:** Rongqin Zhang, Meilinuer Abudurexiti, Wanglin Qiu, Pinbo Huang, Ping Hu, Wei Fan, Zhanwen Zhang

**Affiliations:** 1 Department of Nuclear Medicine, State Key Laboratory of Oncology in South China, Collaborative Innovation Center of Cancer Medicine, Sun Yat-Sen University Cancer Center, Guangzhou, 510060 China; 2 Department of Nuclear Medicine and Molecular Imaging, The Sixth Affiliated Hospital, Sun Yat-sen University, Guangzhou, 510655 China; 3 Department of Nuclear Medicine, The First People’s Hospital of Xinjiang Kashgar Area, Kashgar, Xinjiang 844000 China; 4 Department of Hepatobiliary Surgery, Sun Yat-sen Memorial Hospital, Sun Yat-sen University, Guangzhou, 510120 China

**Keywords:** Incidental colorectal lesions, Positron emission tomography, Colorectal ^18^F-FDG uptake, Water enema

## Abstract

**Background:**

To validate the feasibility of water enema PET/CT (WE-PET/CT) in incidental colorectal ^18^F-FDG uptake and improve the accuracy of diagnosing colorectal neoplastic lesions.

**Methods:**

We retrospectively analysed the electronic records of 338 patients undergoing common PET/CT and WE-PET/CT at our hospital. PET/CT results were correlated with colonoscopy pathology and follow-up results. The ROC contrast curve was plotted to evaluate the accuracy of SUVmax on common PET/CT and WE-PET/CT for detecting neoplastic lesions. SUVmax and the median retention indexes (RIs) of cancerous, precancerous, and benign lesions and physiologic uptake were compared.

**Results:**

The sensitivity, specificity and accuracy of diagnosing neoplastic lesions with common PET/CT were 84.0%, 78.3% and 80.2%, respectively. The corresponding results with WE-PET/CT were 95.8%, 96.5% and 96.2%. The AUC of SUVmax on WE-PET/CT was significantly higher than that on common PET/CT (0.935 vs. 0.524, *p* < 0.001). The median SUVmax on WE-PET/CT was significantly higher than that on common PET/CT in cancerous and precancerous lesions, and significantly decreased in benign lesions and physiologic uptake (*p <* 0.001). The RI was significantly different between cancerous lesions and physiologic uptake, between precancerous lesions and physiologic uptake, between benign lesions and physiologic uptake, and between cancerous and benign lesions (*p* < 0.05).

**Conclusions:**

WE-PET/CT is a noninvasive, well-tolerated and effective technique for diagnosing incidental colorectal ^18^F-FDG uptake. It is helpful for a timely colonoscopy and can effectively avoid an unnecessary colonoscopy for incidental colorectal ^18^F-FDG uptake.

## Background

Combined positron emission tomography/computed tomography (PET/CT) using ^18^F-fluoro-2-deoxy-D-glucose (^18^F-FDG) is an excellent technique for the diagnosis, staging, restaging, and treatment monitoring of various tumours, including colorectal cancers [1]. Incidental focal colorectal ^18^F-FDG uptake is regularly encountered in 1.3–3.4% of patients who undergo PET/CT for reasons other than expected colorectal disease, and it may be associated with inflammatory sites or benign, precancerous or cancerous lesions [2].

Unexpectedly increased ^18^F-FDG uptake in the large bowel can be focal, segmental, or diffuse in pattern [3]. Focal unexpected colorectal ^18^F-FDG uptake might correspond to malignant or premalignant lesions [2, 4, 5], whereas segmental or diffuse colorectal ^18^F-FDG uptake is more frequently associated with physiologic or inflammatory changes [6]. However, it is difficult to differentiate physiologic or benign lesions from neoplastic lesions. Many studies have attempted to differentiate such lesions through various methods, such as analysing the pattern, location, double-phase PET scan and PET parameters such as the maximum standardized uptake value (SUVmax), mean standardized uptake value (SUVmean), metabolic tumour volume (MTV), or total lesion glycolysis (TLG), but a consensus has not yet been reached [2, 7–13]. A subsequent colonoscopy is recommended in patients with colorectal incidental ^18^F-FDG uptake, especially for focal ^18^F-FDG uptake [5, 14, 15]. However, due to the nonspecific ^18^F-FDG uptake of lesions and many false-positive results, some patients may be subjected to unnecessary invasive colonoscopies.

Recently, Kirchner J et al. reported that by taking CT findings in contrast-enhanced CT imaging in terms of wall thickening, intraluminal nodules, and contrast enhancement into account, the specificity of focal colonic ^18^F-FDG uptake for precancerous and cancerous lesions can be increased from 69 to 90% compared with ^18^F-FDG uptake alone but leads to a considerable loss of sensitivity, from 54 to 38% [16]. Although CT findings were taken into consideration, it was still difficult to assess incidental colorectal ^18^F-FDG uptake due to insufficient luminal distension.

Water enema multidetector computed tomography (WE-CT) is a CT technique based on the distension of the colorectum by using water [17]. This technique offers wonderful visualization of the bowel wall, water-filled lumen, surrounding structures and morphological information such as wall-thickening and intraluminal nodules. It provides excellent accuracy for the detection of colorectal cancer and benign lesions [18, 19]. Therefore, we introduced water enema into PET/CT and aimed to precisely diagnose incidental colorectal ^18^F-FDG-avid lesions through WE-PET/CT, correlate WE-PET/CT with subsequent colonoscopy pathology and follow-up results, and evaluate whether WE-PET/CT can provide more sufficient evidence for a timely colonoscopy and help avoid an unnecessary invasive colonoscopy.

## Materials and methods

### Patients

This retrospective study was approved by the ethical committee of our institution (No. 2020ZSLYEC-178). We retrospectively reviewed medical records of patients who had undergone both common PET/CT and WE-PET/CT between January 2010 and December 2019 at our hospital. The inclusion criteria were as follows: (1) patients without known diseases in the location where incidental colorectal ^18^F-FDG uptake was identified, including those who were referred for PET/CT with the diagnosis of a noncolorectal disorder and who had a known colorectal disease but ^18^F-FDG uptake occurred in areas not consistent with the preexisting pathology, (2) patients who underwent an additional water enema scan for incidental colorectal ^18^F-FDG uptake found by common PET/CT, and (3) those in whom a complete colonoscopy was subsequently performed within 6 months or those without positive clinical symptoms, CT and MRI results of colorectum during at least 2-years of follow-up.

### Patient preparation and imaging protocols

Patients were asked to fast for at least 6 h prior to the examination without special bowel preparation. The blood glucose level was controlled at < 10 mmol/L at the time of the injection. Acquisition was performed 50 to 80 min after intravenous injection of 0.1–0.15 mCi/kg of ^18^F-FDG. Imaging was performed using a Biograph True Point 40-slice CT apparatus (TrueD, Siemens Health Care, Erlangen, Germany) or a uMI 780 128-slice CT apparatus (Shanghai United Imaging Healthcare, China) in the supine position. The CT scan (5-mm slice thickness, 120 kV and 200 mA) was performed from the base of the skull to the mid-thigh for attenuation correction and image fusion, followed immediately by a PET scan acquired in three-dimensional mode for 2–3 min per bed position. If incidental colorectal ^18^F-FDG uptake occurred on common PET/CT, WE-PET/CT scans were performed 30 min later using the same parameters. Patient preparation was performed by experienced nuclear medicine nurses. Patients were placed in the left-lateral decubitus position on the PET/CT table, and a lubricated enema tube connected to an enema bag filled with tepid water was gently inserted into the rectum. Then, a certain amount of water was infused through gravity over 2–5 min, 100–200 ml in the rectum, 200–300 ml in the sigmoid colon, 400–500 ml in the descending colon, 500–600 ml in the transverse colon and 600–800 ml in the ascending colon.

### Image interpretation

The ^18^F-FDG PET/CT datasets were independently evaluated by two experienced nuclear medicine doctors. The pattern of colorectal ^18^F-FDG uptake was assessed and localized (ascending colon, transverse colon, descending colon, sigmoid or rectum). The patterns of ^18^F-FDG uptake on PET/CT images were defined as (1) focal: nodular ^18^F-FDG uptake in the colorectum; (2) segmental: sustained area of ^18^F-FDG uptake without sharp borders; and (3) diffuse: area of ^18^F-FDG uptake greater than segmental areas. The intensity of incidental colorectal ^18^F-FDG uptake was quantified as the SUVmax on common PET/CT and WE-PET/CT images. The retention index (RI) was calculated to reflect the change in the SUVmax of colorectal ^18^F-FDG uptake as follows: RI = [SUVmax (WE-PET/CT) – SUVmax (common PET/CT)] / SUVmax (common PET/CT) × 100(%) [20].

The sites of ^18^F-FDG-avid lesions identified were explored by experienced gastroenterologists through colonoscopy within 6 months. Colonoscopy reports were analysed for abnormalities correlating with the incidental finding on the PET/CT scan. When a polypectomy or biopsy of the lesion was recorded, the pathology report combined with an additional histopathological assessment if applicable was subsequently evaluated. The final diagnosis was categorized as follows: cancerous lesions; precancerous lesions (adenomas with or without low or high intraepithelial neoplasia) [21]; benign lesions (including hyperplastic polyps, and inflammatory lesions); and physiologic uptake. Physiologic uptake was defined as ^18^F-FDG uptake seen on common PET/CT with normal colonoscopy, disappearance or morphological changes on WE-PET/CT, no CT findings on WE-PET/CT or no positive findings during follow-up. Except for physiological uptake confirmed by colonoscopy or follow-up, the other three categories were all confirmed by colonoscopy.

On common PET/CT, only ^18^F-FDG uptake was analysed due to insufficient luminal distension. A true positive finding was defined as focal ^18^F-FDG uptake corresponding to cancerous or precancerous lesions on colonoscopy. A false positive finding was defined as focal ^18^F-FDG uptake with a benign lesion or normal finding in colonoscopy. A false-negative finding was defined as segmental, diffuse or no ^18^F-FDG uptake and cancerous or precancerous lesion on colonoscopy.

On WE-PET/CT, both ^18^F-FDG uptake and corresponding CT findings were investigated. A true positive finding was defined as focal ^18^F-FDG uptake with CT findings (wall-thickening or intraluminal nodule) confirmed to be cancerous or precancerous lesions on colonoscopy. A false-positive finding was defined as focal ^18^F-FDG uptake with CT findings and benign lesion or normal finding in colonoscopy. A false-negative finding was defined as segmental, diffuse or no ^18^F-FDG uptake without or with CT findings and cancerous or precancerous lesions on colonoscopy.

### Statistical analysis

Statistical analysis was performed using IBM SPSS version 22 (IBM Inc., Armonk, NY, USA) and MedCalc 15.2 software (MedCalc Software, Mariakerke, Belgium). Quantitative variables are expressed as medians [interquartile ranges, IQRs], and qualitative variables are expressed as numbers and percentages. Mann-Whitney U test was used to compare the SUVmax between neoplastic lesions and benign findings. To evaluate the accuracy of SUVmax on common PET/CT and WE-PET/CT in differentiating neoplastic lesions (cancerous and precancerous lesions) from benign findings (benign lesions and physiologic uptake), an ROC contrast curve was performed and the area under the ROC curve (AUC) was compared by Delong method in MedCalc software. The Wilcoxon’s signed-rank test was used to compare the median SUVmax between common PET/CT and WE-PET/CT for each category. The Kruskal-Wallis test was used to compare the RI among the 4 categories. *P* < 0.05 was considered statistically significant.

## Results

**Fig. 1:**
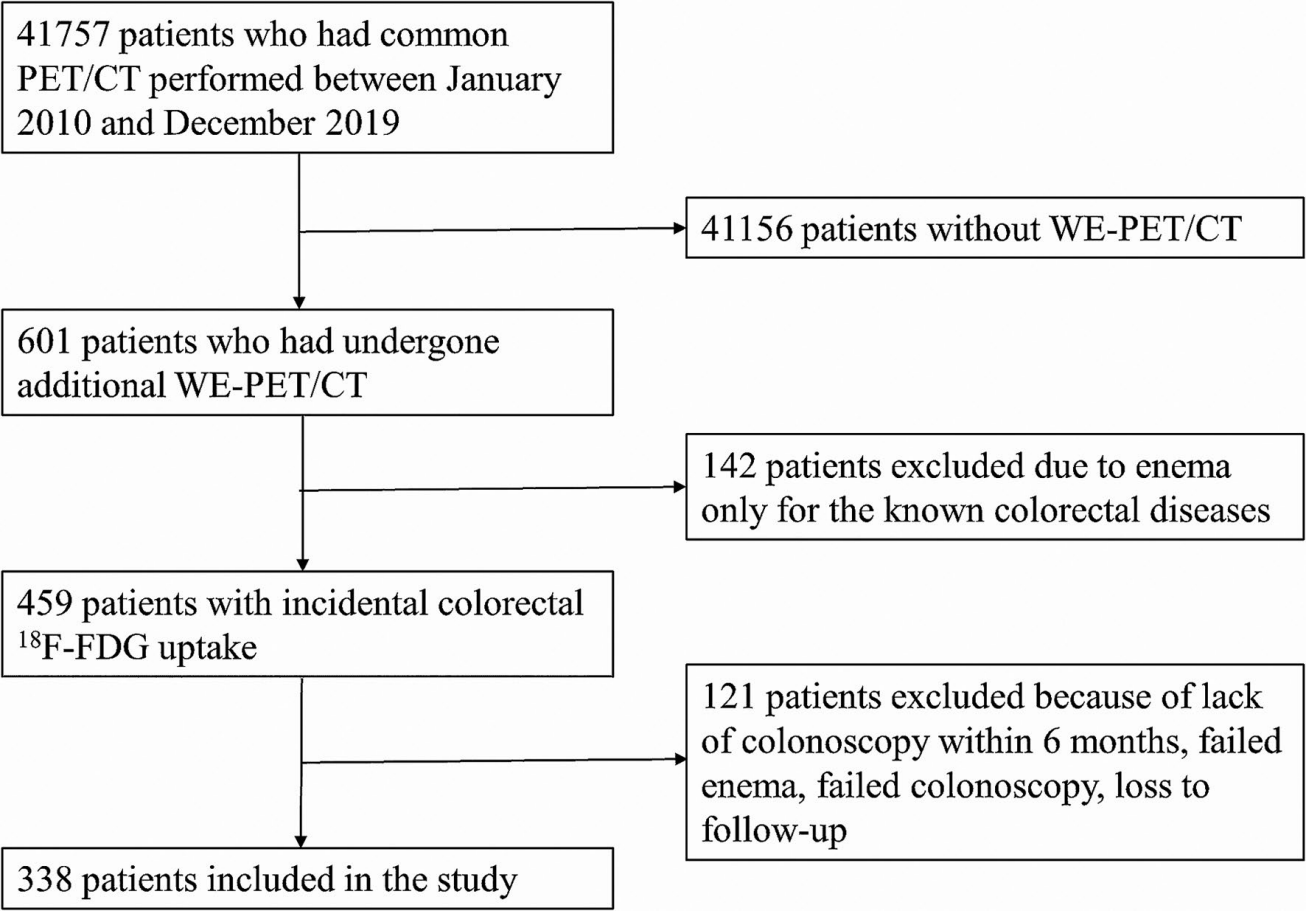
Flowchart of patients

A total of 338 patients with incidental colorectal ^18^F-FDG uptake were included in this study (Fig. 1). There were 219 men and 119 women with a median age of 57 years (range of 19 to 89 years). The indications for PET/CT are shown in Table 1.

**Table 1:** Indications for ^18^F-FDG PET/CT

Scan indication	Number of patients	Percentage (%)
Metastatic cancer of unknown origin	24	7.1
Lung cancer	29	8.6
Colorectal carcinoma	48	14.2
Head and neck cancer	24	7.1
Cancer screening	107	31.7
Suspicion of inflammatory disease	7	2.1
Gastric cancer	22	6.5
Lymphoma	14	4.1
Gynecologic cancer	21	6.2
Esophageal cancer	8	2.4
Genitourinary cancer	7	2.1
Breast cancer	4	1.2
Hepatopancreatobiliary cancer	10	3.0
Others	13	3.8
Total	338	100

## PET/CT results and colonoscopic findings

In 39.1% (132/338) of patients, focal colorectal ^18^F-FDG uptake was observed within 155 lesions, while in 20.7% (70/338) of patients, segmental ^18^F-FDG uptake was observed, and in 40.2% (136/338) of patients, diffuse ^18^F-FDG uptake was observed.

**Fig. 2:**
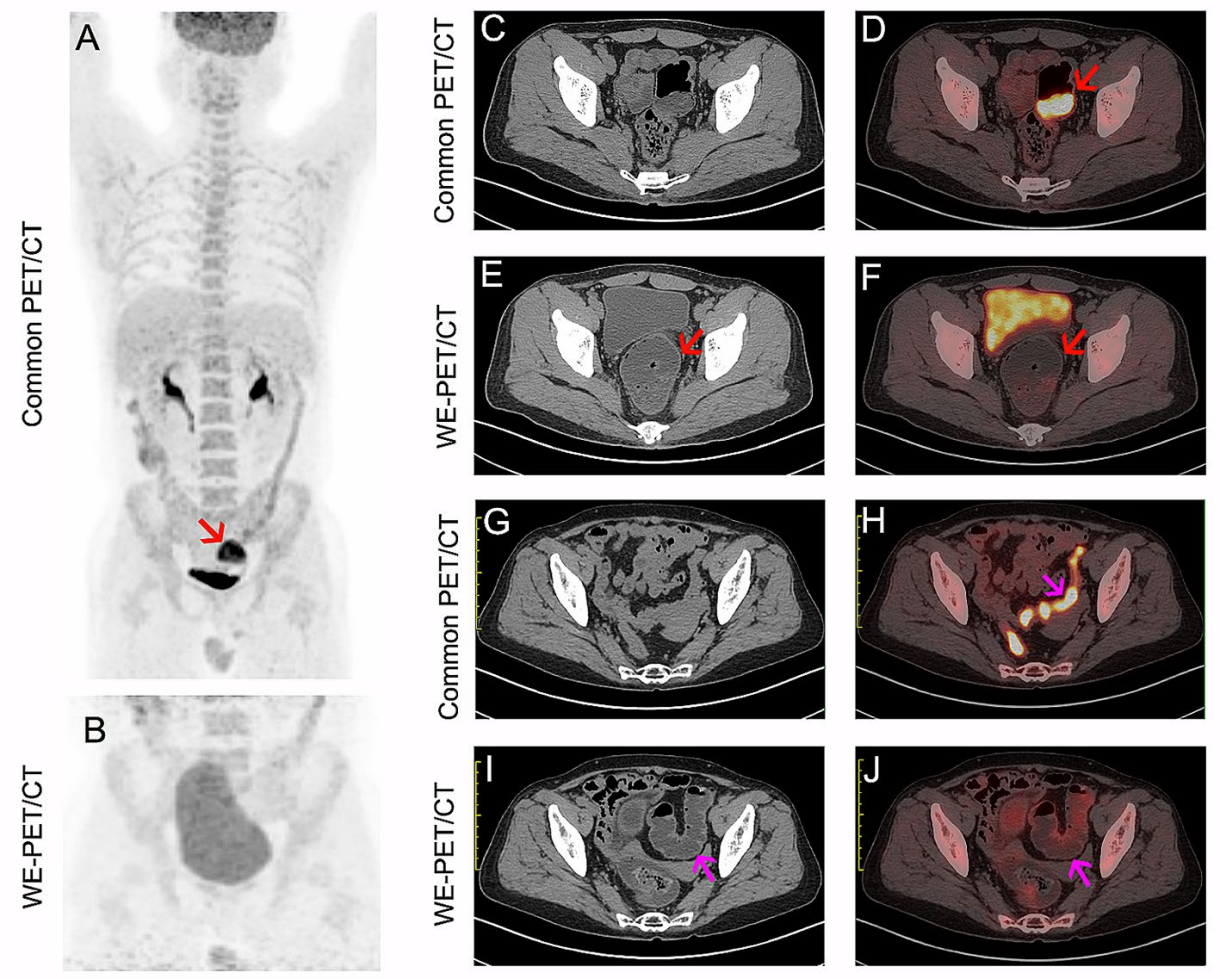
Representative image of focal (**A**-**F**, red arrow) and segmental (**G**-**J**, pink arrow) physiologic ^18^F-FDG uptake. The ^18^F-FDG uptake on common PET/CT (**A**, **D** and **H**) disappeared after water enema (**B**, **F** and **J**)

**Fig. 3:**
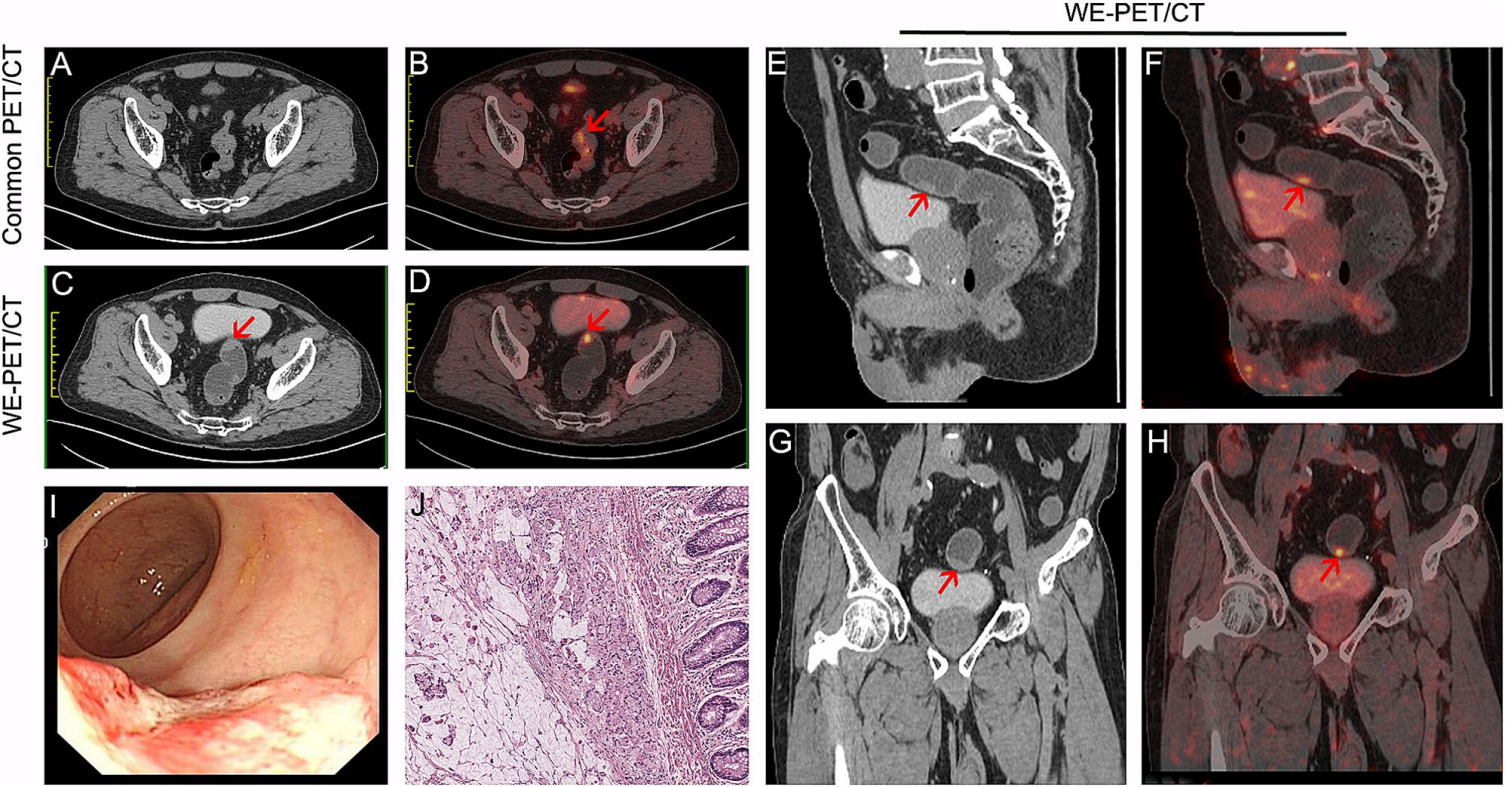
A 74-year-old male patient with cancer screening. Uneven ^18^F-FDG uptake in the sigmoid colon and a collapsed bowel was shown on common PET/CT (**A**-**B**). On WE-PET/CT, the tumour was clearly shown as focal ^18^F-FDG uptake with correlative wall thickening (**C**-**H**, red arrow). Colonoscopy (**I**) and pathological analysis (**J**) confirmed T1 mucinous adenocarcinoma

**Fig. 4:**
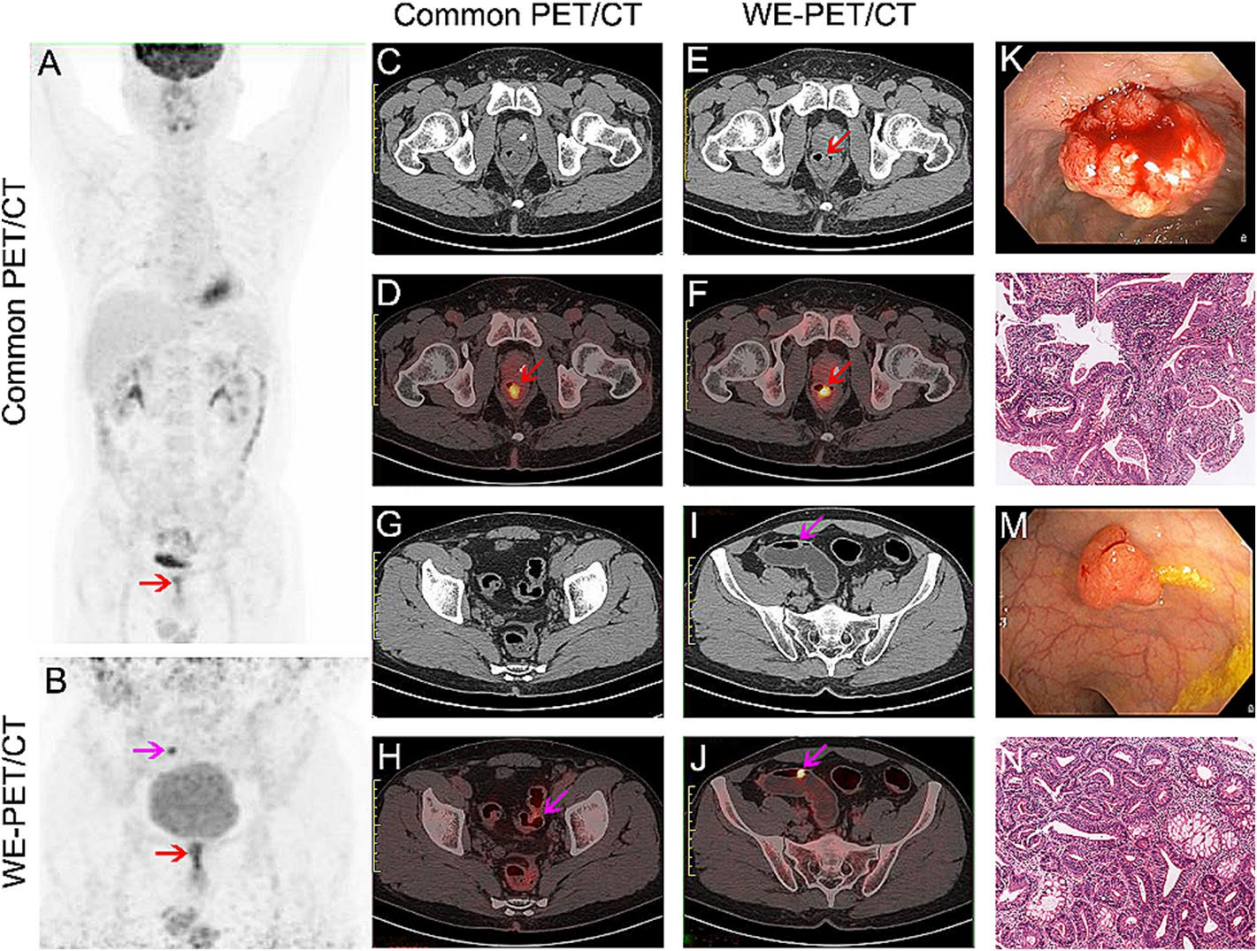
A 46-year-old male patient undergoing cancer screening. One incidental focal ^18^F-FDG uptake in the rectum was observed on common PET/CT (**A**, **C**-**D**). Two intraluminal nodules with ^18^F-FDG uptake in the rectum and sigmoid were detected on WE-PET/CT (**B**, **E**-**F** and **I**-**J**). The colonoscopy and pathological analysis confirmed tubulovillous adenoma of the rectum (**K**, **L**) and tubular adenoma with high-grade intraepithelial neoplasias of the sigmoid (**M**, **N**)

Of 132 patients with focal colorectal ^18^F-FDG uptake, 116 had a single focus of colorectal ^18^F-FDG uptake, 11 patients had two foci, 4 patients had three foci, and 1 patient had five foci. The distribution of foci on PET/CT was as follows: ascending colon (n = 13), transverse colon (n = 7), descending colon (n = 12), sigmoid colon (n = 81), and rectum (n = 42). Of the 155 focal unexpected ^18^F-FDG accumulations, 46 (29.7%) showed physiologic ^18^F-FDG uptake (Fig. 2A-F). In the remaining 109 sites, cancerous lesions were diagnosed in 58 (37.4%, Fig. 3), precancerous lesions in 42 (27.1%, Fig. 4) and benign lesions in 9 (5.8%), as shown in Table 2.

**Table 2:** Relationship of the ^18^F-FDG uptake pattern and colonoscopy pathology and follow-up results

Lesion	n	^18^F-FDG Uptake			
		Focal	Segmental	Diffuse	No uptake
Cancerous lesions	66	58	3	5	0
Precancerous lesions					
High grade	10	9	1	0	0
Low grade	43	33	4	1	5
Benign lesions					
Hyperplastic polyp	12	4	0	1	7
Inflammatory lesions	28	5	14	9	0
Physiologic uptake	214	46	48	120	0
Total	373	155	70	136	12

**Fig. 5:**
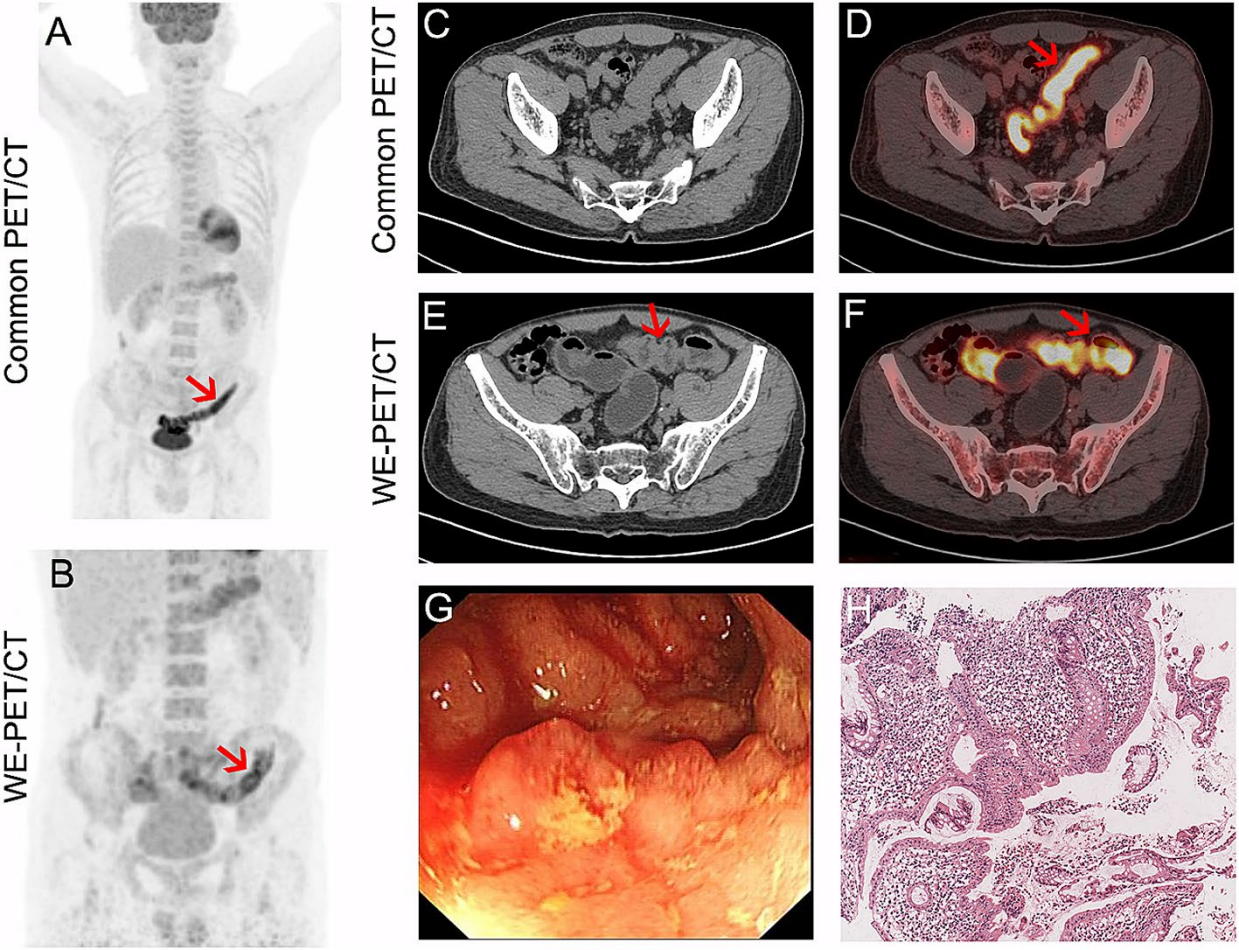
A 61-year-old male patient with IgG4-related pancreatitis. Segmental ^18^F-FDG uptake in the sigmoid was observed on common PET/CT (**A**, **C** and **D**). The ^18^F-FDG uptake in the sigmoid was reduced on WE-PET/CT, with mild and uniform wall thickening (**B**, **E** and **F**, red arrow). The colonoscopy (**G**) and pathological analysis (**H**) confirm ulcerative colitis

Of the 70 patients with unexpected segmental ^18^F-FDG accumulation, 68.6% (48/70) of patients exhibited physiologic ^18^F-FDG uptake (Fig. 2G-J). Cancerous, precancerous, and benign lesions were present in 3, 5 and 14 patients, respectively (Fig. 5).

**Fig. 6:**
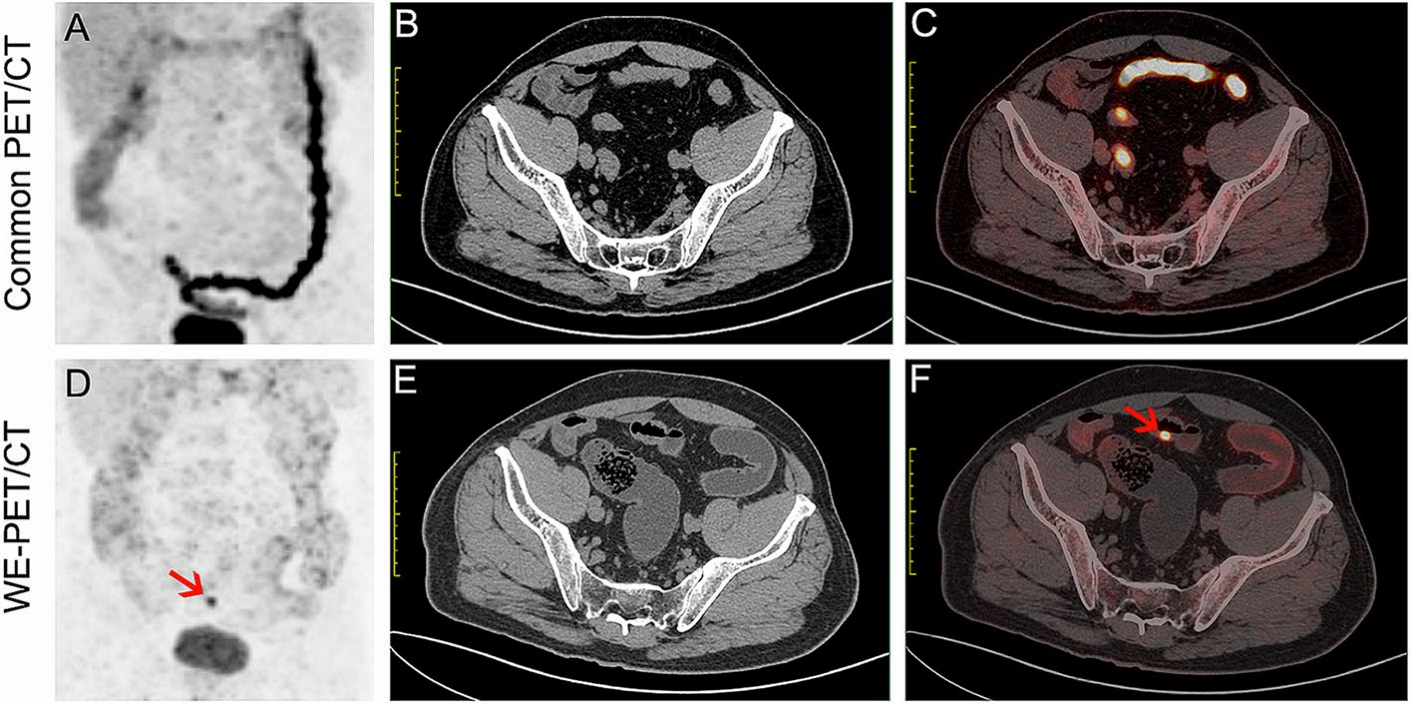
A small lesion hidden in diffuse ^18^F-FDG uptake. Strong diffuse ^18^F-FDG uptake in the colorectum with a collapsed bowel is shown on the CT scan (**A**-**C**). After water enema, diffuse ^18^F-FDG uptake disappeared except for a focal ^18^F-FDG-avid lesion in the sigmoid with localized wall thickening (**D**-**F**, red arrow)

Of the 136 patients with diffuse unexpected ^18^F-FDG accumulation, 88.2% (120/136) presented physiologic ^18^F-FDG uptake. Cancerous lesions, precancerous lesions and benign lesions were present in 5, 1 and 10 patients, respectively. These small lesions were hidden in diffuse ^18^F-FDG uptake (Fig. 6).

Additionally, 5 low-grade adenomas (0.3–0.8 cm in diameter) and 7 hyperplastic polyps (0.3–0.5 cm in diameter) were incidentally detected by colonoscopy. These lesions did not show any ^18^F-FDG uptake on either common PET/CT or WE-PET/CT.

### Diagnostic performance of common PET/CT and WE-PET/CT

The overall sensitivity, specificity, accuracy, positive predictive value (PPV) and negative predictive value (NPV) for the detection of colorectal neoplastic lesions with common PET/CT were 84.0% (100/119), 78.3% (199/254), 80.2% (299/373), 64.5% (100/155) and 91.3% (199/218), respectively. On WE-PET/CT, five small adenomas were missed due to lack of any ^18^F-FDG uptake, and 4 hyperplastic polyps and 5 inflammatory lesions had been diagnosed as false positive due to focal ^18^F-FDG uptake and corresponding CT findings, leading to a sensitivity, specificity, accuracy, PPV and NPV of 95.8% (114/119), 96.5% (245/254), 96.2% (359/373), 92.7% (114/123) and 98.0% (245/250), respectively.

**Fig. 7:**
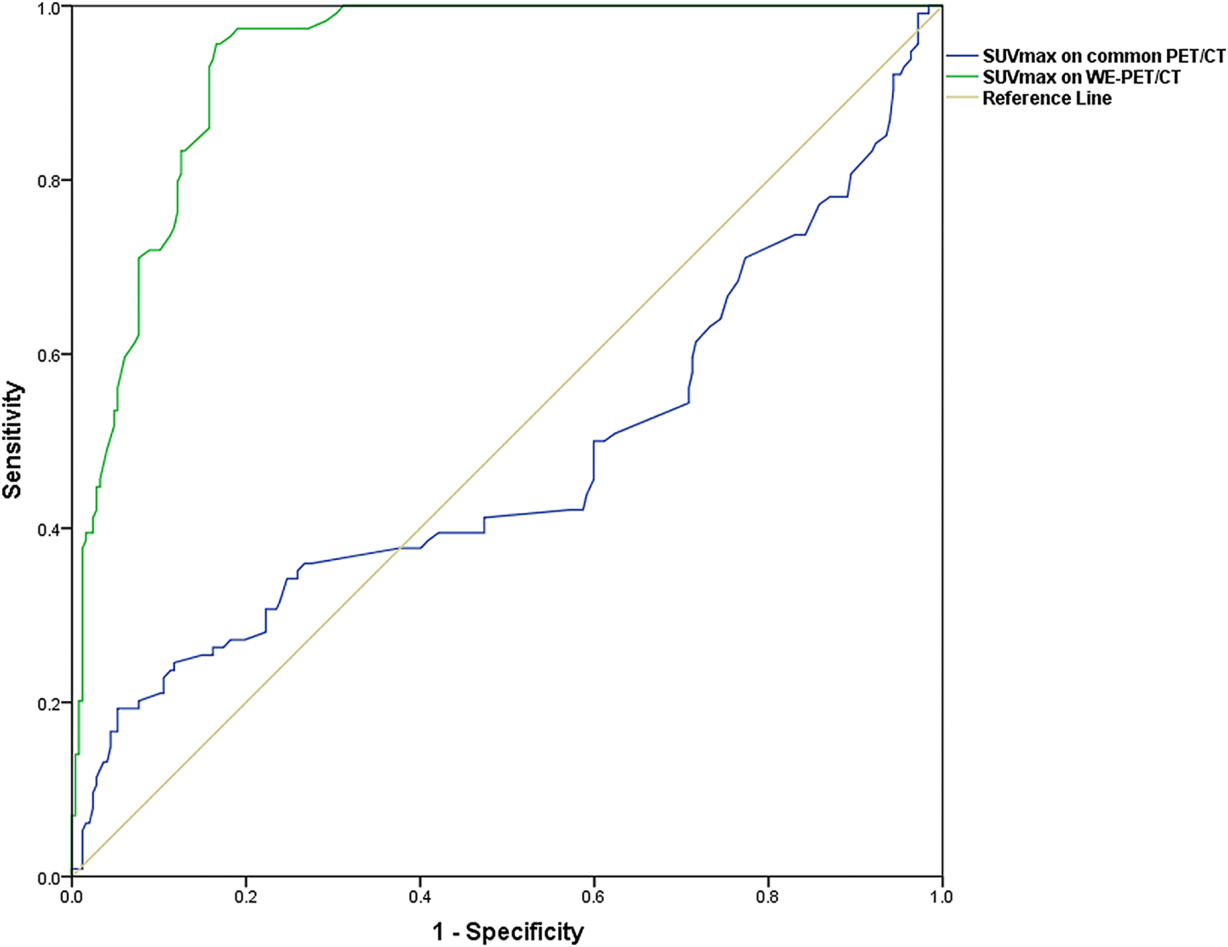
Comparisons of the AUC of SUVmax on common PET/CT and WE-PET/CT in differentiating neoplastic lesions and benign findings

On common PET/CT, there was no significant difference in the median SUVmax between neoplastic lesions and benign findings (7.3[5.3] vs. 8.0[3.1], *p* = 0.459). However, on WE-PET/CT the median SUVmax of neoplastic lesions was significantly higher than that of benign findings (7.6[5.7] vs. 2.4[1.8], *p <* 0.001). The AUC of SUVmax on WE-PET/CT was significantly higher than that on common PET/CT in differentiating neoplastic lesions from benign findings (0.935 vs. 0.524, *p* < 0.001, Fig. 7). In ROC curve analysis, SUVmax > 4.3 on WE-PET/CT predicted neoplastic lesions with a sensitivity of 95.6% and specificity of 83.4%.

### Analysis of SUVmax and RI on common PET/CT and WE-PET/CT

Based on the within-group comparison, the median SUVmax on WE-PET/CT was significantly higher than that on common PET/CT in cancerous lesions (7.5[6.0] vs. 7.0[6.4], *p* = 0.001) and precancerous lesions (7.7[4.9] vs. 7.4[4.8], *p* = 0.02). However, compared to common PET/CT, the median SUVmax on WE-PET/CT significantly decreased in benign lesions (7.7[4.0] vs. 6.0[4.2], *p <* 0.001) and physiologic uptake especially (8.0[3.1] vs. 2.0[1.5], *p* < 0.001).

**Fig. 8:**
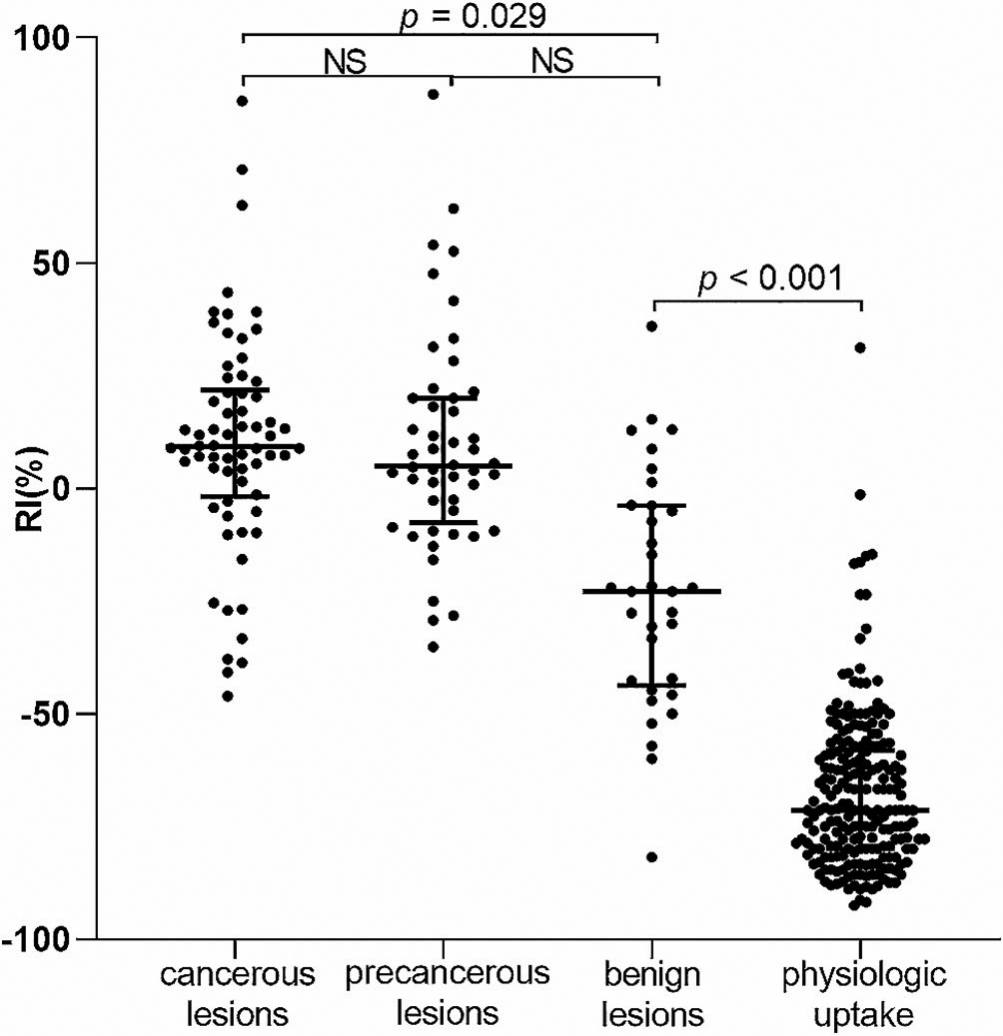
Comparison of the RI among the four groups. The error bar indicates the median [IQR]. NS: no significance

The median RIs in benign cancerous, precancerous, benign lesions and physiological uptake were 9.21[23.59], 4.99[27.70], -22.83[39.93] and − 71.43[21.92], respectively. The between-group comparison showed that there was a significant difference in the RIs between cancerous lesions and physiologic uptake (*p* < 0.001), between precancerous lesions and physiologic uptake (*p* < 0.001), between benign lesions and physiologic uptake (*p* < 0.001), and between cancerous and benign lesions (*p* = 0.029). No significant difference in RI was observed between cancerous and precancerous lesions, or between precancerous and benign lesions (Fig. 8).

## Discussion

Incidental colorectal ^18^F-FDG uptake is a relatively common finding in patients undergoing PET/CT. However, it is challenging to diagnose colorectal ^18^F-FDG uptake in clinical practice. In attempts to distinguish malignant from benign lesions, different PET parameters have been analysed with little success to date [2, 7, 10]. Although CT scans have great advantages in exploring lesion characteristics, such as size, shape, density and location, the colonic cavity is collapsed on common PET/CT, which is a nonnegligible factor increasing the difficulty of diagnosis. Luminal distension is a fundamental requirement for CT imaging of the bowel because collapsed segments may hide the presence of tumours or polypoid lesions. WE-CT can fully expand the colorectal cavity and easily show thickened intestinal walls and lesion characteristics. The present study investigated the effectiveness of the WE-PET/CT imaging technique in incidental colorectal ^18^F-FDG uptake. In this study, WE-PET/CT revealed a sensitivity of 95.8% and specificity of 96.5% for diagnosing neoplastic lesions, significantly higher than the reported sensitivity of 38% and specificity of 90% based on common PET/CT and contrast-enhanced CT imaging [16].

Although colonoscopy is regarded as the recommended standard for colorectal lesions, it has shortcomings in terms of cost, safety, dietary preparation and bowel cleansing, and patient comfort and acceptance are rather low [8, 16, 22]. PET/CT colonography, using water or air for colonic distension, has been applied for imaging colorectal polyps and colorectal cancers [23–25]. From the standpoint of patient-related feasibility, Gollub et al. reported that 94% (17/18) of patients tolerated scanning and Taylor et al. demonstrated that patients preferred PET/CT colonography over colonoscopy [24, 26]. In our study, more than 90% of patients tolerated WE-PET/CT well, including elderly patients (44% of patients older than 60 years). WE-PET/CT may be a satisfactory alternative noninvasive examination, especially for elderly individuals.

Many previous studies have demonstrated that diffuse and segmental patterns often represent physiologic or inflammatory uptake, while focal ^18^F-FDG uptake usually indicates a high risk of cancerous or precancerous lesions, and a further colonoscopy is warranted; thus, patients with nonfocal ^18^F-FDG uptake were not evaluated further [2, 14]. In our evaluated sites, 31.4% (22/70) of segmental and 11.8% (16/136) of diffuse unexpected colorectal ^18^F-FDG uptake corresponded to a structural abnormality identified on colonoscopy. Furthermore, 36.4% (8/22) and 37.5% (6/16) of lesions were cancerous or precancerous. Water enema can eliminate the interference of physiologic uptake, which is beneficial for detecting small lesions hidden in diffuse and segmental colorectal ^18^F-FDG uptake. Previous studies have shown that 13.7-56% of incidental focal ^18^F-FDG uptake lesions on PET/CT had no correlative colonic lesion on colonoscopy [2, 4, 27, 28]. In our study, 29.7% (46/155) of focal ^18^F-FDG uptake was actually physiologic uptake, consistent with the above findings. The exact mechanisms of physiologic uptake are unclear; however, physiologic smooth muscle activation, a metabolically active mucosa, swallowed secretions, or colonic microbial uptake are presumed [29, 30]. After water enema, physiologic ^18^F-FDG uptake in the colorectum declined dramatically. We speculate that the considerable amount of water diluted physiologic ^18^F-FDG excretion in the colorectum. It is not reliable to differentiate neoplastic lesions from benign findings by uptake pattern only because high segmental and diffuse ^18^F-FDG uptake may hinder the detection of such lesions, while intense focal ^18^F-FDG uptake may result in false positives. Compared to common PET/CT, WE-PET/CT provides more sufficient evidence for a further colonoscopy, and can effectively avoid unnecessary further colonoscopies.

Semiquantitative analysis of ^18^F-FDG avidity was performed by calculating the SUVmax for diagnosing incidental colorectal lesions. However, the relationship between the SUVmax and histopathologic features of colorectal lesions is controversial. Luboldt et al. suggested an SUVmax cutoff of > 5 for differentiating malignant from nonmalignant lesions [31]. Oh JR et al. and Van Hoeij et al. proposed a higher SUVmax cutoff of 9.1 and 11.4 for a high risk of malignancy, respectively, and a colonoscopy should be performed without delay [2, 8]. However, we observed 18 lesions with an SUVmax less than 5 related to cancerous or precancerous lesions, while 47 lesions with an SUVmax more than 11.4 corresponded to normal findings on colonoscopy. In contrast, some studies have shown that the SUVmax is not discriminative enough to distinguish cancerous, precancerous and benign lesions, and the SUVmax of colorectal ^18^F-FDG uptake on common PET/CT shows significant overlap between neoplastic lesions and benign findings [16, 20, 32], which is in accordance with our results. These results indicate that the SUVmax on common PET/CT should be considered with caution to differentiate neoplastic lesions and benign findings. Simsek FS et al. examined the utility of dual-time-point common ^18^F-FDG PET/CT and observed that neither SUVmax nor RI are reliable for detecting colorectal neoplastic lesions, which was inconsistent with our results since we used WE-PET in the delayed phase [13]. In our study, the SUVmax of 72.7% cancerous lesions and 62.5% of precancerous lesions increased after water enema. The reason may be mainly due to the delayed imaging nature of WE-PET/CT. In contrast to neoplastic lesions, the SUVmax of benign findings significantly decreased after water enema. Our study shows that SUVmax on WE-PET/CT not on common PET/CT and RIs may provide some differential diagnostic value, and a further SUVmax > 4.3 on WE-PET/CT predicted neoplastic lesions with a sensitivity of 95.6% and specificity of 83.4%.

Although neither SUVmax nor RIs were sufficient to differentiate between cancerous and precancerous lesions, the lesion morphological characteristics on WE-PET/CT may provide some reference values. Most cancerous lesions show intense ^18^F-FDG uptake on both common PET/CT and WE-PET/CT, accompanied mostly by focal wall thickening or intraluminal nodules on the WE-PET/CT scan, while most precancerous lesions show intraluminal nodules. In contrast to neoplastic lesions, the RIs were significant in separating benign lesions from physiologic uptake. Inflammatory lesions are presumably ^18^F-FDG-avid on PET/CT because of the increased metabolism of inflammatory cells [5]. Hyperplastic polyps are usually considered benign nonneoplastic lesions of the colorectum, and their metabolic behavior is controversial. Kamel EM et al. and Keyzer C et al. reported that 3 and 9/26 hyperplastic polyps were ^18^F-FDG-avid respectively, while Ravizza, D et al. and Abdel-Nabi H et al. reported that none of the 26 and 35 hyperplastic polyps tended to accumulate ^18^F-FDG, respectively [12, 33–35]. In our study, 5 of the 12 hyperplastic polyps showed positive ^18^F-FDG uptake, and we speculate that this may be related to proliferative activity. Of the 42 adenomas detected by focal ^18^F-FDG uptake, 6 were less than 1 cm in diameter, of which 5 were low-grade and one was high-grade adenomas histologically. Additionally, 5 small low-grade adenomas (0.3–0.8 cm) were not ^18^F-FDG-avid. Igarashi K et al. reported that sensitivity of PET to advanced adenomas (≥ 1 cm in size) and low-grade adenomas (0.6–0.9 cm) was 50.7% and 9.3%, respectively [36]. Therefore, histologic grade and size were the most important factors affecting adenoma visibility at PET.

There are two limitations in this study. First, it is a retrospective study in a single institution and the results might have been influenced by selection bias. Second, most patients with physiologic ^18^F-FDG uptake in the colorectum did not undergo colonoscopy, especially those with disappeared ^18^F-FDG uptake after water enema, leading to failure to detect small-sized non-^18^F-FDG-avid adenomas or polyps. The non-^18^F-FDG-avid adenomas were classified as precancerous lesions in our study, so the sensitivity of WE-PET/CT in diagnosing neoplastic lesions was actually overestimated. However, lack of ^18^F-FDG uptake might exclude the presence of a lesion with an actual clinical impact, and further there were no clinical and imaging positive findings during in these patients at least 2-years of follow-up.

## Conclusion

WE-PET/CT is a safe, noninvasive, well-tolerated and effective imaging technique. It seems to show high sensitivity, specificity and accuracy for diagnosing incidental colorectal ^18^F-FDG uptake, effectively avoid unnecessary colonoscopies and provide sufficient evidence for further necessary colonoscopies. WE-PET/CT not only effectively reduces the interference of physiologic ^18^F-FDG uptake in the colorectum but also correctly characterizes all cases of colorectal focal ^18^F-FDG uptake. Cancerous and precancerous lesions on WE-PET/CT can be effectively distinguished from benign lesions and physiologic ^18^F-FDG uptake than on common PET/CT.

## Data Availability

The data that support the findings of this study are available from the corresponding author upon reasonable request. The data are not publicly available due to information that could compromise the privacy of research participants.
